# Oral Delivery of Liraglutide Formulated with PLGA for Sustained Obesity Management

**DOI:** 10.3390/ijms27073300

**Published:** 2026-04-05

**Authors:** Nipeng Chen, Zhipeng Zeng, Xiaoyu Ji, Weijia Huang, Zhen Zhang, Yongming Chen

**Affiliations:** 1 PCFM Lab, Guangdong Engineering Technology Research Centre for Functional Biomaterials, School of Materials Science and Engineering, Sun Yat-sen University, Guangzhou 510006, China; chennp@mail2.sysu.edu.cn (N.C.); jixy9@mail2.sysu.edu.cn (X.J.); huangwj79@mail2.sysu.edu.cn (W.H.); 2 Guangxi Key Laboratory of Special Biomedicine, School of Medicine, Guangxi University, Nanning 530004, China; zengzhp25@mail.sysu.edu.cn; 3 State Key Laboratory of Biocontrol, Guangdong Provincial Key Laboratory of Plant Stress Biology, School of Life Sciences, Sun Yat-sen University, Guangzhou 510006, China; 4 State Key Laboratory of Molecular Engineering of Polymers, Fudan University, Shanghai 200437, China; 5 State Key Laboratory of Advanced Polymer Materials, Sichuan University, Chengdu 610207, China; 6 State Key Laboratory of Antiviral Drugs, College of Chemistry and Molecular Science, Henan University, Zhengzhou 450046, China

**Keywords:** obesity, liraglutide, oral delivery, nanovesicle

## Abstract

Liraglutide (Lira), a glucagon-like peptide-1 (GLP-1) receptor agonist, has demonstrated substantial efficacy in improving glycemic control and reducing body weight. However, subcutaneous injection is poorly adherent for patients. To improve treatment compliance, we developed a poly(lactic-co-glycolic acid) (PLGA)-based nanovesicle (PLGA-Lira-NV) system for the oral delivery of Lira using a double-emulsion solvent evaporation technique. The optimized formulation yielded a narrow size distribution and high encapsulation efficiency (>95%). In vitro release studies showed that PLGA-Lira-NVs remained relatively stable under acidic conditions (pH 1.2 to 6.8) and exhibited sustained drug release in a neutral environment (pH 7.4), enabling protection of the fragile peptide in the stomach and controlled release after crossing the intestine. Following oral administration to obese mice (10 mg/kg), PLGA-Lira-NVs achieved prolonged glycemic control for up to 72 h. Notably, body weight decreased to 83% of baseline after 12 days, outperforming the subcutaneous injection (free Lira) group (88%). The consistent trend toward weight reduction confirms the sustained-release properties of PLGA nanocarrier for Lira, highlighting its potential to reduce dosing frequency and improve patient compliance. Collectively, these findings underscore the promising potential of PLGA nanovesicles as an oral delivery platform for peptide therapeutics.

## 1. Introduction

Obesity, a chronic metabolic disorder characterized by excessive fat accumulation, has emerged as a major global health crisis. By 2030, the number of obese adults worldwide is expected to exceed 1.1 billion, with over half of the world’s population likely to be overweight or obese by 2035. This epidemic is no longer confined to high-income countries, but is rapidly expanding across low- and middle-income nations, driven by urbanization, reduced physical activity, and increased consumption of energy-dense foods with low nutritional value. The growing burden of obesity is not detrimental to individual health, but also imposes significant economic and social costs, underlining the urgent need for effective obesity management. Glucagon-like peptide-1 (GLP-1) receptor agonists, such as liraglutide (Lira), are currently the main drugs used clinically for weight management [1]. However, subcutaneous administration is associated with local pain and potential risk of infection, which urgently requires the development of novel delivery systems to enhance therapeutic efficacy and patient acceptance [2,3,4].

Compared to subcutaneous injection, oral administration has clinical advantages such as easy operation, safety, and painlessness, which are suitable for long-term treatment of chronic diseases [5]. In addition, the production of oral pharmaceuticals requires fewer aseptic conditions, and its storage and transportation process can get rid of the dependence on cold chain facilities. However, oral administration of peptide/protein therapeutics faces significant challenges due to the presence of multiple biological barriers (including biochemical, mucus, and epithelial barrier) in the digestive system [6]. Recent breakthroughs in oral delivery have provided effective solutions to improve the bioavailability of peptide/protein therapeutics, with nanotechnology being particularly prominent. By means of physical encapsulation or chemical modification, nanocarriers have significantly enhanced the stability of peptide drugs and facilitated trans-epithelial absorption [7,8]. Although various nanocarrier systems, such as liposomes [9], polymeric micelles [10], dendrimers [11], and gold nanoparticles [12] have demonstrated moderate potential to improve oral delivery efficiency, uncontrolled release kinetics, potential carrier toxicity, complex fabrication processes, and limited access to scalable production hinder their clinical translations.

**Figure 1:**
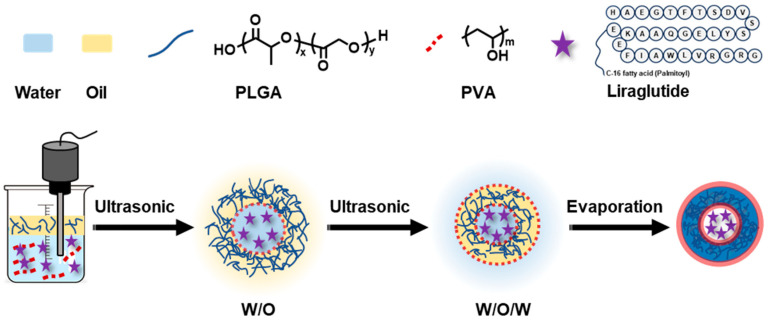
Schematic of liraglutide-loaded PLGA nanovesicle preparation by ultrasonic-assisted double emulsion method. The oil phase is ethyl acetate.

Herein, an oral delivery system based on poly(lactic-co-glycolic acid) (PLGA, a classically degradable polymer approved by the FDA) for Lira therapeutics was developed. Up to now, PLGA-based systems have attracted considerable attention due to their biodegradability and ability to protect peptide drugs from degradation. Ismail et al. reported Lira-loaded PLGA nanoparticles and showed that encapsulation improved enzymatic stability under simulated gastrointestinal conditions and increased Lira permeability across a Caco-2 intestinal model [13]. In parallel, several PLGA-based Lira formulations have been developed primarily as injectable sustained-release systems for diabetes treatment, aiming to prolong drug release, reduce injection frequency, and improve glycemic control [14]. More recently, oral Lira nanocarriers have also been explored to enhance intestinal transport, mucus penetration, and hypoglycemic efficacy, but these studies have largely been conducted in diabetes-related models and mainly focused on oral absorption or glucose regulation [15,16,17,18]. By contrast, orally delivered PLGA-encapsulated Lira, specifically developed and evaluated for obesity or body-weight management, remains relatively underexplored. In this context, the present study aims to develop an oral PLGA-Lira nanovesicle system and investigate its potential as a noninvasive Lira delivery strategy for weight management. Lira-loaded PLGA nanovesicle was prepared by a facile and efficient double-emulsion solvent evaporation method. Specifically, an aqueous solution of liraglutide and polyvinyl alcohol (PVA) was mixed with an organic phase (ethyl acetate) containing PLGA by ultrasonic emulsification to form a water-in-oil (W/O) primary emulsion. This emulsion was subsequently dispersed into an external PVA aqueous phase to form a water-in-oil-in-water (W/O/W) double emulsion, followed by solvent evaporation to form PLGA nanovesicles, as illustrated in Figure 1. Critical physicochemical parameters, including particle size distribution, surface charge, encapsulation efficiency, and in vitro stability, were systematically optimized. In vivo studies demonstrated that PLGA-Lira-NV had sustained glycemic control and significant weight reduction.

## 2. Results and Discussion

### 2.1. Preparation of PLGA Vesicles via Double Emulsion Method

The ultrasonic-assisted solvent evaporation method is a well-established and effective approach for fabricating PLGA-based vesicles. As illustrated in Figure 1, PLGA is first dissolved in an organic solvent (ethyl acetate) to form the oil phase. This oil phase is emulsified with an aqueous phase containing polyvinyl alcohol (PVA) as an emulsifier through ultrasonication, generating a primary water-in-oil (W/O) emulsion. The primary emulsion is then subjected to a second emulsification step, in which it is dispersed into another emulsifier-containing aqueous phase to form a water-in-oil-in-water (W/O/W) double emulsion. Subsequently, under gentle heating and rotary evaporation, the ethyl acetate is gradually removed, leading to the solidification of PLGA and the formation of vesicles with the drug encapsulated within their cores. The key advantage of this method lies in the application of ultrasound, which induces strong cavitation effects that efficiently reduce droplet size and enhance the uniformity of particle distribution, resulting in nanovesicles with smaller and more homogeneous sizes. Moreover, the double emulsion system provides a multiphase architecture with increased interfacial surface area and additional encapsulation space. Both the internal aqueous and oil phases can serve as drug reservoirs, enabling the incorporation of hydrophilic and hydrophobic drugs. Compared to traditional single-emulsion systems, the W/O/W structure significantly improves hydrophilic drug loading capacity and encapsulation efficiency, making it particularly suitable for peptide or protein delivery applications.

**Figure 2:**
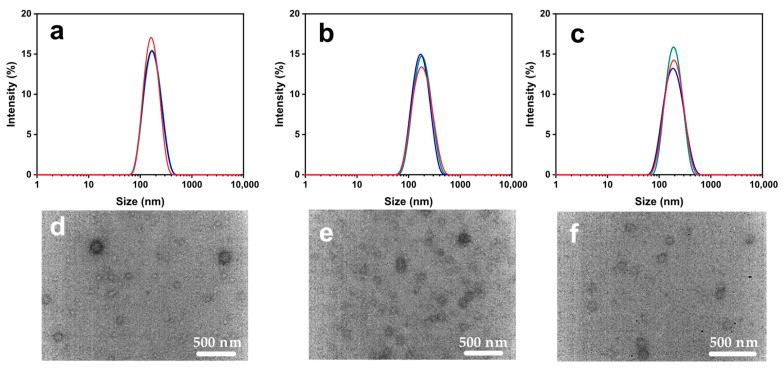
Particle size distribution of empty nanovesicles prepared with PLGA concentrations of (**a**) 10 mg/mL, (**b**) 20 mg/mL, and (**c**) 30 mg/mL (each measured in triplicate). TEM images of blank nanovesicles at corresponding PLGA concentrations: (**d**) 10 mg/mL, (**e**) 20 mg/mL, and (**f**) 30 mg/mL.

PLGA concentration plays a pivotal role in vesicle formation, which prompted us to investigate the preparation of empty nanovesicles at different PLGA concentrations. As shown in Figure 2 and Figure S1, the average vesicle diameter measured by DLS in aqueous solution increased slightly with increasing PLGA concentration, reaching approximately 114 ± 5 nm at 10 mg/mL (Figure 2a), 118 ± 2.3 nm at 20 mg/mL (Figure 2b), and 127 ± 1 nm at 30 mg/mL (Figure 2c), which consistent with the TEM results in Figure 2d,e. This trend could be attributed to the progressive thickening of the vesicle membrane as PLGA content increases, leading to larger particle sizes. Notably, the polydispersity index (PDI) decreased significantly as PLGA concentration increased. This phenomenon may be associated with the shell-forming role of PLGA in the nanovesicles. A higher PLGA concentration is likely to facilitate the formation of a more uniform and robust vesicle shell, which in turn improves the homogeneity of the nanovesicles and leads to a narrower size distribution. In addition, vesicles fabricated via sonication with those produced by high-speed magnetic stirring were compared. As shown in Figures S2 and S3, stirring-generated PLGA vesicles (PLGA-NV-L) displayed significantly larger particle sizes (average diameter about 1.8 ± 0.2 μm by TEM), clearly demonstrating the superior capability of ultrasonication in producing uniformly sized nanovesicles.

### 2.2. Characterization and In Vitro Lira Release of PLGA-Lira-NV

**Figure 3:**
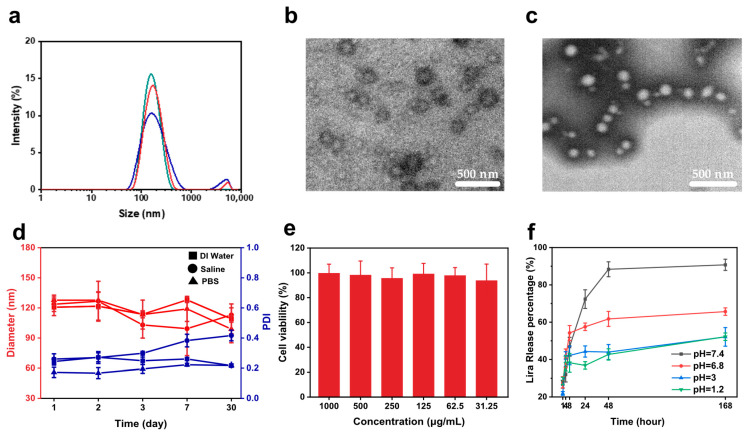
(**a**) Particle size distribution of PLGA-Lira-NV (PLGA concentration: 30 mg/mL). (**b**) TEM image of PLGA-Lira-NV without negative staining. (**c**) TEM image of PLGA-Lira-NV with negative staining. (**d**) Stability assessment of PLGA-Lira-NV in water, saline, and PBS, showing changes in particle size and PDI over 30 days. Data are presented as mean ± standard deviation (*n* = 3). (**e**) Dose–response curves of Caco-2 cell viability following treatment with varying concentrations of PLGA-Lira-NV. (**f**) In vitro release profile of Lira from PLGA-Lira-NV in double-distilled water at pH 1.2, 3.0, 6.8, and 7.4.

After optimizing the preparation conditions for blank PLGA nanovesicles, drug-loaded PLGA-Lira-NV was fabricated by substituting the aqueous phase in the primary emulsion step with a 10 mg/mL Lira solution, using PLGA dissolved in 28 mg/mL PVA as the organic phase. The nanovesicles were then prepared via the double-emulsion solvent evaporation method. The resulting formulation was characterized, and the results are presented in Figure 3a. The average particle size of PLGA-Lira-NV was 138 ± 5 nm, slightly larger than that of the unloaded vesicles (127 ± 3 nm), and the zeta potential was measured to be −0.6 mV. To determine drug loading, the nanovesicle suspension was subjected to ultrafiltration, and the concentration of unencapsulated Lira in the supernatant was quantified using a bicinchoninic acid (BCA) protein assay. Encapsulation efficiency (EE) and drug loading capacity (LC) were calculated using Equations (1) and (2), yielding values of 95.7% and 11.1%, respectively. The high encapsulation efficiency is more reasonably interpreted as arising from the double-emulsion-based encapsulation process and the effective confinement of Lira within the aqueous core. After negative staining with phosphotungstic acid, which enhances contrast by depositing high electron density tungsten atoms onto the low-density nanostructure surfaces, TEM images revealed well-defined, uniformly distributed vesicles with enhanced structural clarity for PLGA-Lira-NV compared to blank vesicles, as shown in Figure 3b,c. Colloidal stability was assessed by dispersing PLGA-Lira-NV in distilled water, phosphate-buffered saline, and saline, followed by monitoring particle size over 30 days (Figure 3d). The PDI in normal saline exceeded 0.35 after 7 days, indicating a little aggregation, while size uniformity was well-maintained in both phosphate-buffered saline and saline throughout the study period. This stability is mainly attributed to the steric stabilization provided by PVA during formulation, while the nonionic polymeric nature of PVA gives rise to a nearly neutral surface charge on the nanovesicles.

The in vitro cytotoxicity of PLGA-Lira-NV was evaluated using Caco-2 cells and the CCK-8 assay. As shown in Figure 3e, cell viability remained above 90% across all tested concentrations (15.6–1000 μg/mL), demonstrating low cytotoxicity and favorable biocompatibility. The pH-dependent drug release behavior of PLGA-Lira-NV was further investigated in simulated gastrointestinal conditions (pH 1.2, 3.0, 6.8, and 7.4) at 37 °C (Figure 3f). The release rate was highest at pH 7.4, with 72% of Lira released within 24 h and reaching 90% at 168 h. At pH 6.8, an initial burst release (54% at 4 h) was followed by a slower release phase, achieving 65% at 168 h. In contrast, release in acidic media was significantly slower, with only 42% and 37% released within 4 h at pH 3.0 and pH 1.2, respectively, and cumulative release plateauing at approximately 52% after 168 h. These findings confirm that PLGA-Lira-NV exhibits a pH-responsive release profile, with faster release under alkaline conditions, consistent with PLGA’s accelerated degradation at higher pH [19,20,21]. The stability under acidic conditions (pH 1.2–3.0) protects the drug from premature release in the gastric environment, thereby improving oral bioavailability. In contrast, the enhanced release in intestinal conditions (pH 6.8–7.4) ensures efficient and site-specific drug delivery. To further elucidate the release mechanism, the in vitro release profiles of PLGA-Lira-NV at different pH values were fitted using zero-order, first-order, Higuchi, Korsmeyer–Peppas, and Weibull models. The results showed that the release behavior did not follow a simple zero-order process. Under pH 6.8 and 7.4 conditions, the Weibull model provided the best fit, indicating a biphasic release pattern with an initial burst followed by sustained release, suggesting the combined involvement of drug diffusion and gradual PLGA matrix relaxation/degradation. In contrast, under acidic conditions, particularly at pH 1.2, the release profile was better described by the Higuchi and Korsmeyer–Peppas models, indicating predominantly diffusion-controlled release. In addition, the release exponent n from the Korsmeyer–Peppas model was below 0.43, supporting Fickian diffusion as the dominant mechanism during the initial stage (Table S1). These results further support the pH-responsive release behavior of PLGA-Lira-NV. This sustained and controlled release behavior is advantageous for reducing dosing frequency, minimizing plasma concentration fluctuations, and enhancing patient adherence.

### 2.3. PLGA Vesicle Transports Across the Intestinal Mucus Layer

**Figure 4:**
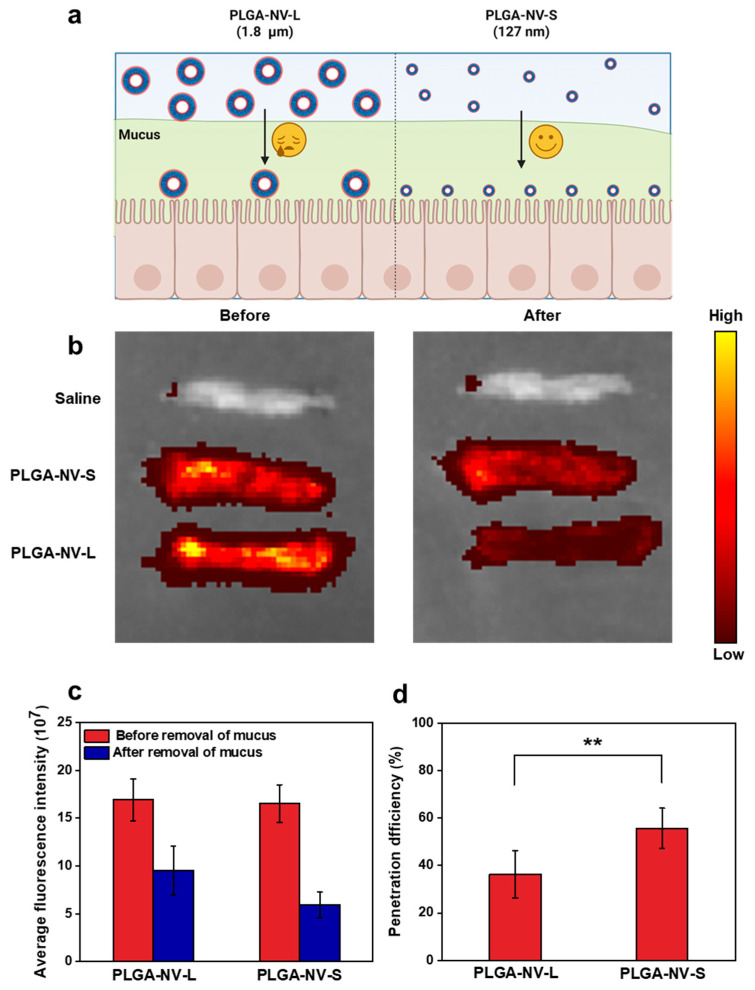
(**a**) Schematic illustrating that small-sized PLGA-NVs (127 ± 3 nm) exhibit superior mucus penetration efficiency compared to their larger counterparts (1.8 ± 0.3 μm). (**b**) Representative in vitro fluorescence images of jejunal tissues treated with Cy7-OVA-labeled nanovesicles, shown before and after mucus layer removal. (**c**) Quantification of average fluorescence intensity in jejunal tissue. (**d**) Mucus-penetration efficiency of nanovesicles. Statistical analyses were conducted using GraphPad Prism V.8.0 and SPSS V.25. Specifically, these software packages were used to calculate the mean values and standard deviations and to generate the figures. Statistical markers indicate significant differences between groups. (** *p* < 0.01). Data are presented as mean ± standard error.

The intestinal mucosal surface is covered by a mucus layer that functions as a protective barrier against external pathogens and harmful substances. However, this layer also represents a major obstacle for orally administered drugs, which must penetrate it to reach systemic circulation. In this study, a small intestinal loop model was employed to investigate the distribution and permeation behavior of drug-loaded PLGA nanovesicles (labeled with Cy7-OVA) within the intestinal mucus layer (Figure 4a). As shown in Figure 4b, the fluorescence distribution of three groups was compared: large-sized PLGA nanovesicles (PLGA-NV-L, prepared via high-speed stirring), small-sized PLGA nanovesicles (PLGA-NV-S, prepared using an ultrasonic homogenizer), and a saline control group, both before and after removal of the mucus layer. Figure 4c presents the quantitative analysis of mean fluorescence intensity in the small intestine. Before mucus removal, both PLGA-NV-L (1.8 ± 0.2 μm) and PLGA-NV-S (127 ± 3 nm) exhibited strong fluorescence signals (1.69 × 10^8^ and 1.65 × 10^8^, respectively). However, following mucus removal, fluorescence intensity decreased to 9.54 × 10^7^ for PLGA-NV-S and to 5.92 × 10^7^ for PLGA-NV-L, indicating a more substantial reduction for the larger nanovesicles. These findings suggest that larger particles experience greater resistance in traversing the mucus barrier. The intestinal mucus layer is composed of a porous, mesh-like network formed by mucin glycoproteins, with pore sizes ranging approximately from 100 to 500 nm [22,23]. Nanovesicles with smaller sizes are better able to avoid physical entrapment within this mesh, thereby achieving more efficient penetration. As calculated in Figure 4d, the mucosal penetration efficiency was 55.7% for PLGA-NV-S and 36.3% for PLGA-NV-L, confirming the size-dependent permeation capacity. In addition to size, surface charge, and hydrophilicity also play crucial roles in mucus penetration. The mucus layer is negatively charged and tends to retain positively charged particles through electrostatic interactions, which reduces their diffusivity [24]. The surface potential of PLGA-NV-S was measured at −0.6 mV, a near-neutral and slightly negative charge that may facilitate diffusion through the negatively charged mucus, thereby enhancing the penetration efficiency of the hydrophilic, drug-loaded vesicles across the intestinal mucosa.

### 2.4. The Hypoglycemic and Anti-Obesity Effects of PLGA-Lira-NV

**Figure 5:**
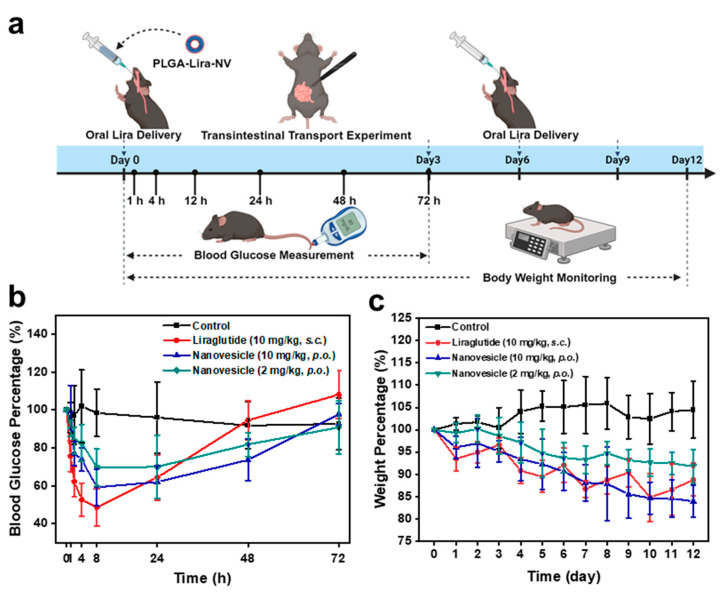
(**a**) Schematic representation of the in vivo experimental schedule. Blood glucose concentrations in mice were assessed at 1, 4, 8, 24, 48, and 72 h after dosing. Anti-obesity interventions were performed on days 0, 3, 6, and 9, with body weight changes recorded daily for 12 days after the initial treatment. The figure was created with BioRender.com (**b**) Blood glucose level fluctuations in obese mice following treatment with liraglutide via subcutaneous injection (10 mg/kg) and oral administration of PLGA-Lira-NV at low (2 mg/kg) and high doses (10 mg/kg). (**c**) Body weight changes in obese mice across different PLGA-Lira-NV treatment groups throughout the study.

To evaluate the therapeutic efficacy of orally administered PLGA-Lira-NV in an obese mouse model, this study first investigated its ability to sustain blood glucose reduction following a single administration (Figure 5a). As detailed in Section 3, mice were randomly assigned to four groups: control, subcutaneous injection, high-dose oral administration (10 mg/kg), and low-dose oral administration (2 mg/kg). Each group received either a single subcutaneous injection of Lira or a single oral gavage of PLGA-Lira-NV. As shown in Figure 5b, the subcutaneous injection group exhibited a rapid hypoglycemic response, with blood glucose levels dropping to 73% of baseline within 1 h post-administration and reaching a nadir of 48% at 8 h, followed by a sharp rebound. In contrast, both oral groups showed a more gradual onset of action. Blood glucose levels reached their lowest points at 8 h post-dose as well, 59% in the high-dose group and 70% in the low-dose group, but the hypoglycemic effect was maintained for up to 72 h, indicating a prolonged therapeutic duration. To further assess anti-obesity efficacy, body weight changes were monitored across the four groups. Based on the observed 72 h duration of the glucose-lowering effect, the oral treatment groups were dosed every 3 days until day 12. As depicted in Figure 5c, the control group exhibited continuous weight gain, reaching 105% of initial body weight by day 12 under ad libitum feeding. In contrast, all treatment groups showed significant weight reduction. By day 12, the subcutaneous injection group (10 mg/kg) reduced to 88% of baseline weight, the high-dose oral group (10 mg/kg) to 83%, and the low-dose oral group (2 mg/kg) to 92%. Notably, while the subcutaneous group experienced a transient weight rebound after day 2, the high-dose oral group achieved a more sustained and consistent weight loss trajectory. Furthermore, even at one-fifth the dose, the low-dose oral group exhibited a significant and stable anti-obesity effect. Collectively, these findings suggest that oral PLGA-Lira-NV provides a prolonged glucose-lowering effect and demonstrates promising potential as an oral delivery system for Lira. While direct superiority over conventional injectable therapy cannot be concluded without further pharmacokinetic and dose-normalized studies, this strategy represents a potentially attractive alternative for obesity management due to its sustained pharmacodynamic profile and improved convenience of administration.

### 2.5. In Vivo Safety Evaluation

**Figure 6:**
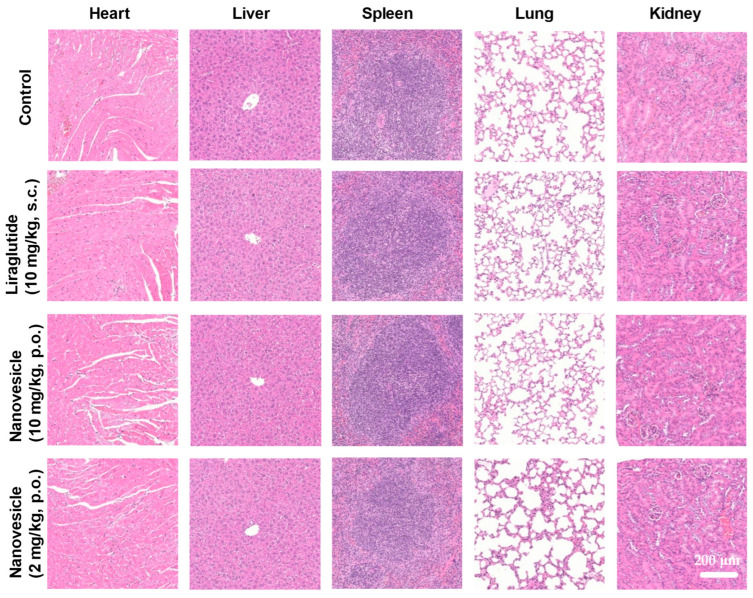
Histological examination results of major organs stained with H&E for different PLGA-Lira-NV treatment groups.

As shown in Figure 6, the in vivo safety evaluation of PLGA-Lira-NV demonstrated no significant pathological changes in major organs, including the heart, liver, spleen, lungs, and kidneys. Furthermore, no signs of inflammation or histopathological abnormalities were observed in the skin at the administration sites. There were no significant differences in tissue morphology compared to the control group. Comprehensive histological analyses confirmed that both subcutaneous injection of free Lira and oral administration of PLGA-Lira-NV (at both high and low doses) preserved the structural integrity and normal histoarchitecture of all examined organs. These results indicate that PLGA-Lira-NV possesses excellent biocompatibility and does not induce systemic or local toxicity, supporting its potential for safe therapeutic application in the treatment of obesity and related metabolic disorders.

## 3. Materials and Methods

### 3.1. Materials

Liraglutide and polyvinyl alcohol (PVA-1788) were purchased from Aladdin (Shanghai, China). PLGA (lactide: glycolide is 50:50, *M*_w_ is 90,000) and ethyl acetate were purchased from Macklin (Shanghai, China). Cy7-labeled ovalbumin (Cy7-OVA) was obtained from Abcam (Shanghai, China).

### 3.2. Preparation and Characterization of PLGA-Lira-NV

The preparation of PLGA-Lira-NV involved dispersing 1 mL of a PVA aqueous solution (28 mg/mL) containing 10 mg/mL Lira into 3 mL of an oil phase (ethyl acetate) with 30 mg/mL PLGA using an ultrasonic homogenizer under ice bath conditions at 75% power for 90 s of continuous sonication to form the primary W/O emulsion [25]. The primary emulsion was then added to 10 mL of a PVA aqueous solution (20 mg/mL) under the same ultrasonic conditions to form a double W/O/W emulsion. Finally, ethyl acetate was removed via rotary evaporation, and the product was freeze-dried to obtain PLGA-Lira-NV. Under identical preparation conditions, 1 mL of the PVA aqueous solution (28 mg/mL) was replaced with an equal volume of a PVA solution containing 0.2 mg/mL Cy7-labeled ovalbumin (Cy7-OVA), yielding PLGA vesicles loaded with Cy7-OVA.

To investigate the effect of vesicle size on intestinal mucosal penetration efficiency, large-sized PLGA vesicles were prepared via high-speed stirring. Specifically, 3 mL of an ethyl acetate solution containing 30 mg/mL PLGA was placed in a small flask with a magnetic stir bar, and 1 mL of a PVA aqueous solution (28 mg/mL) was slowly added dropwise under stirring at 1200 rpm at room temperature for 2 min. The primary emulsion was then gradually introduced into 10 mL of a PVA aqueous solution (20 mg/mL) under the same stirring conditions to form a double emulsion. After rotary evaporation to remove ethyl acetate, large-sized PLGA vesicles (PLGA-NV-L) with a zeta potential of −0.9 ± 0.3 mV were obtained. The colloidal stability of PLGA-Lira-NV was assessed by dispersing the nanovesicles in distilled water, phosphate-buffered saline (PBS), and saline, and subsequently monitoring changes in particle size over 30 days.

The size, zeta potential, and polydispersity index (PDI) of the PLGA-Lira-NV were characterized using a Malvern Zetasizer NanoZS90 (Malvern Instruments Ltd., Malvern, UK). The morphology of PLGA-Lira-NV was tested via transmission electron microscopy (TEM, JEOL-1400, JEOL Ltd., Tokyo, Japan). A 4 mL sample of PLGA-Lira-NV was transferred into a 15 mL ultrafiltration tube (Macklin, Shanghai, China, molecular weight cutoff 30 kDa). The tube was centrifuged at 5000 rpm for 20 min in a high-speed centrifuge. The concentration of free Lira in the ultrafiltrate was quantified using a BCA protein assay kit (Thermo Fisher Scientific, Shanghai, China). The encapsulation efficiency (EE) and drug loading capacity (LC) of the PLGA-Lira-NV sample were calculated using Equations (1) and (2), respectively.

(1) $$\mathrm{EE}\;(\%)=\frac{\mathrm{weight}\;\mathrm{of}\;\mathrm{Lira}\;\mathrm{in}\;\mathrm{nanoparticle}}{\mathrm{total}\;\mathrm{weight}\;\mathrm{of}\;\mathrm{drug}}\times 100\%$$

(2) $$\mathrm{LC}\;(\%)=\frac{\mathrm{weight}\;\mathrm{of}\;\mathrm{Lira}\;\mathrm{in}\;\mathrm{nanoparticle}}{\mathrm{total}\;\mathrm{weight}\;\mathrm{of}\;\mathrm{nanoparticle}}\times 100\%$$

### 3.3. Freeze-Drying and Storage of PLGA-Lira-NV

The PLGA-Lira-NV solution was transferred into a dialysis bag (molecular weight cutoff = 100 kDa) and dialyzed against water overnight. The dialyzed PLGA-Lira-NV solution was then aliquoted into centrifuge tubes and freeze-dried at room temperature for 48 h under a vacuum pressure of 10 Pa. The resulting PLGA-Lira-NV powder was sealed and stored at −80 °C until further use. Before characterization, the lyophilized powder was reconstituted in double-distilled water, and its particle size, Zeta potential, and PDI were measured using a Malvern laser particle size analyzer.

### 3.4. Cytotoxicity Evaluation of PLGA-Lira-NV

Before in vivo studies, the cytotoxicity of PLGA-Lira-NV was assessed using Caco-2 cells. After 24 h of cell culture, predetermined concentration gradients of PLGA-Lira-NV suspension (1000, 500, 250, 125, 62.5, and 31.25 μg/mL) were added to the cells in 100 μL volumes, followed by an additional 24 h of incubation. Cell viability was then determined by adding CCK-8 reagent and measuring the absorbance using a microplate reader. This assay allowed for the determination of PLGA-Lira-NV’s biocompatibility at different concentrations, providing critical safety data before proceeding with in vivo experiments. The CCK-8 assay, which measures cellular metabolic activity via mitochondrial dehydrogenase activity, served as a reliable indicator of cell viability and potential cytotoxic effects.

### 3.5. In Vitro Drug Release

To simulate the pH changes experienced by nanovesicles during oral administration as they pass through the gastrointestinal tract (stomach pH 1.2–3, small intestine pH 6.8–7.4), in vitro release experiments were conducted at pH 1.2, 3, 6.8, and 7.4. The experimental procedure was as follows: The PLGA-Lira-NV solution was first concentrated 10-fold using an ultrafiltration tube. Then, 1 mL of the concentrated solution was transferred into a dialysis bag with a molecular weight cutoff of 100 kDa. The sealed dialysis bag was immersed in a centrifuge tube containing 15 mL of distilled water adjusted to the target pH (1.2, 3, 6.8, or 7.4). The system was incubated in a shaking incubator at 37 °C and 150 rpm. At predetermined time points (1, 4, 8, 24, 48, and 168 h), 1 mL of the release medium was withdrawn for analysis, and an equal volume of fresh buffer at the corresponding pH was immediately added to maintain sink conditions. The Lira concentration in the collected samples was quantified using a BCA protein assay kit. This pH-dependent release study provided critical insights into the drug release behavior of PLGA-Lira-NV under physiological conditions, particularly how the nanoparticle formulation responds to the varying acidity levels encountered during gastrointestinal transit. The dynamic exchange system ensured accurate measurement of drug release kinetics while maintaining constant experimental conditions.

### 3.6. Animal Experiment

The animal experiment was conducted following the “Guidelines for the Care and Use of Laboratory Animals” and adhered to the protocols approved by the Institutional Animal Care and Use Committee (IACUC) at Sun Yat-sen University (Application No. 2024-0195). Female C57BL/6 mice, aged 6 weeks, were obtained from the Animal Experiment Center of Sun Yat-sen University.

### 3.7. Establishment of a Mouse Model of Obesity

To induce obesity in mice, a high-fat diet was employed. Female C57BL/6 mice, known for their sensitivity to high-calorie intake and a high modeling rate, were selected. The diet comprised 60 kcal% fat (HF60, Guangdong Provincial Animal Center). Mice in the experimental group were provided with a high-fat diet, while those in the control group received a standard diet. Weekly body weight measurements were recorded to compare weight differences between obese and normal mice. The model was deemed successfully established when the body weight of obese mice exceeded 30% of that of the normal mice, which typically occurred after approximately 10 weeks.

### 3.8. Animal Grouping and Administration Protocol

Obesity-induced mice established via a high-fat diet were randomly divided into three treatment groups and one control group, with five mice per group. The treatment groups were further categorized into: subcutaneous injection group receiving 10 mg/kg free Lira, high-dose oral administration group receiving PLGA-Lira-NV equivalent to 10 mg/kg Lira, and low-dose oral administration group receiving PLGA-Lira-NV at a 2 mg/kg Lira dose. For oral gavage, mice were first restrained, and a 46 mm gastric feeding needle was carefully inserted through the oral cavity into the esophagus, with an insertion depth of 3–4 cm (to avoid tracheal injury).

### 3.9. Intestinal Mucosal Transport Study

C57 mice (*n* = 3 per group) were fasted for 15 h to empty the intestinal tract. Under anesthesia, a 5 cm segment of jejunum was isolated, ligated at both ends to create a closed loop, and injected with 0.5 mL of Cy7-OVA-loaded PLGA nanovesicles. After 2 h, the intestinal segment was excised, longitudinally opened, and rinsed three times with PBS. Fluorescence imaging was first performed on the intact segment using an in vivo imaging system. The mucus layer was then removed via vacuum suction, followed by a second fluorescence imaging session. Quantitative analysis was conducted by comparing the mean fluorescence intensity before and after mucus removal to calculate the permeability rate.

### 3.10. Hypoglycemic Effect of PLGA-Lira-NV

The blood glucose-lowering effect was evaluated by monitoring glycemic changes post-administration. Mice were grouped and dosed as described in Section 3.8. Blood samples were collected via tail vein puncture at 1, 4, 8, 24, 48, 72, and 96 h post-dosing. After discarding the first drop of blood, 20 μL of blood was collected using a glucometer to measure and record blood glucose levels.

### 3.11. The Anti-Obesity Efficacy of PLGA-Lira-NV and In Vivo Safety Evaluation

The anti-obesity effect was assessed by monitoring body weight changes in treated mice. Mice were grouped and dosed as outlined in Section 3.2. Administration was performed every three days for a total of four treatments. During the experimental period, mice had ad libitum access to a high-fat diet, with daily recordings of individual body weight and food intake. Results were compared with those of age-matched normal mice (20–22 g) that were not induced to be obese. The in vivo safety of PLGA-Lira-NV was evaluated in major organs, including the heart, liver, spleen, lungs, and kidneys. The histomorphological changes were evaluated by hematoxylin and eosin staining.

## 4. Conclusions

In this study, a PLGA-based nanovesicle system was developed to address the challenges of oral peptide delivery. As a proof of concept, a Lira-loaded PLGA nanovesicle was prepared by a double-emulsion evaporation method. The resulting nanovesicle was featured with facile preparation, controlled particle size, resistance to stomach acid, and effective mucus penetration. Moreover, it also demonstrated sustained hypoglycemic effects and significant weight reduction after oral administration, outperforming in terms of efficacy and duration in comparison to subcutaneous injection of liraglutide at the same dose (10 mg/kg). Notably, the nanovesicles achieved moderate therapeutic efficacy even with low-dose (2 mg/kg) oral administration, suggesting a dose-dependence in weight control. These findings highlight the potential of PLGA nanovesicles as a non-invasive, sustained-release oral platform for peptide therapeutics in chronic disease management, and this oral delivery strategy provides a promising solution to overcome bioavailability limitations of peptide therapeutics, offering enhanced therapeutic outcomes and patient convenience for long-term obesity management.

## Data Availability

The raw data supporting the conclusions of this article will be made available by the authors on request.

## Supplementary Materials

The following supporting information can be downloaded at: https://www.mdpi.com/article/10.3390/ijms27073300/s1.
