# Supplementary material for: Oral Delivery of Liraglutide Formulated with PLGA for Sustained Obesity Management
Source: Int J Mol Sci. 2026 Apr 5;27(7):3300. doi: 10.3390/ijms27073300 (PMC13072843; doi:10.3390/ijms27073300)
Supplement: Supplementary file 1 [file ijms-27-03300-s001.zip › ijms-4218889-supplementary.pdf]

Supplementary data

Oral Delivery of Liraglutide Formulated with PLGA for Sustained Obesity Management

Nipeng Chen [1,†], Zhipeng Zeng [2,†], Xiaoyu Ji [1], Weijia Huang [1], Zhen Zhang [1,3,4,5,*] and Yongming Chen [1,6,*]

[1] 1 PCFM Lab, Guangdong Engineering Technology Research Centre for Functional Biomaterials, School of Mate-rials Science and Engineering, Sun Yat-sen University, Guangzhou 510006, China;

2 Guangxi Key Laboratory of Special Biomedicine, School of Medicine, Guangxi University, Nanning 530004, China;
3 State Key Laboratory of Biocontrol, Guangdong Provincial Key Laboratory of

Plant Stress Biology, School of Life Sciences, Sun Yat-sen University, Guangzhou 510006, China
4 State Key Laboratory of Molecular Engineering of Polymers, Fudan University, Shanghai 200437, China
5 State Key Laboratory of Advanced Polymer Materials, Sichuan University, Chengdu 610207, China
6 State Key Laboratory of Antiviral Drugs, College of Chemistry and Molecular Science, Henan University, Zhengzhou 450046, China
* Correspondence: zhangzh379@mail.sysu.edu.cn (Z. Zhang); chenym35@mail.sysu.edu.cn (Y. Chen)
† These authors contributed equally to this work.

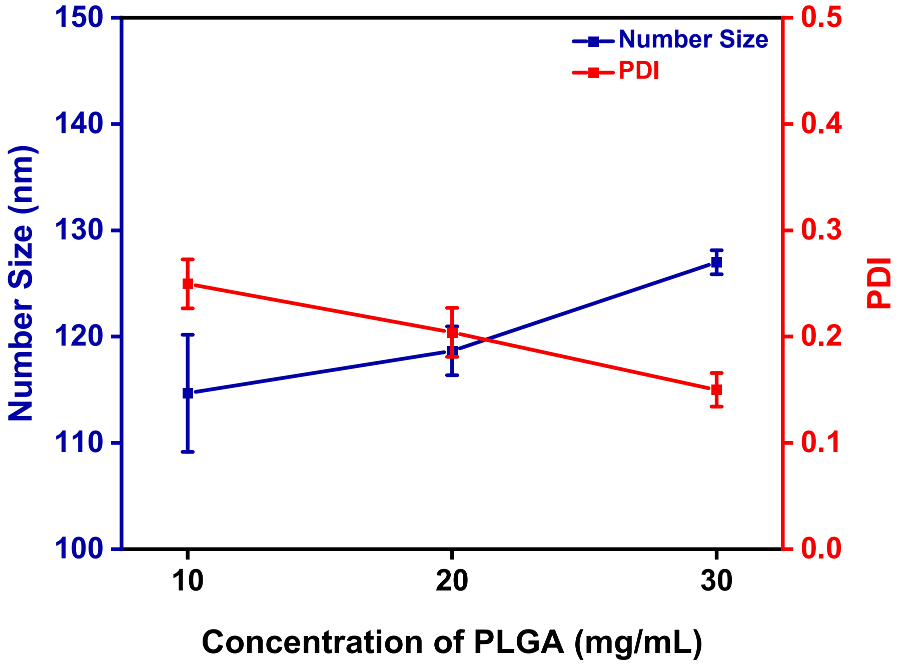

Figure S1. The effect of PLGA concentration on the particle size and PDI of PLGA-Lira-NV.

Table S1. Kinetic modeling of liraglutide release from PLGA-Lira-NV under different pH conditions.

pH	Zero-order	First-order	Higuchi	Korsmeyer–Peppas		Weibull	Suggested mechanism
	R^2	R^2	R^2	R^2	n	R^2	
7.4	0.551	0.823	0.772	0.862	0.286	0.962	Biphasic release; initial diffusion followed by matrix relaxation/degradation
6.8	0.436	0.576	0.647	0.896	0.213	0.888	Burst plus sustained release; diffusion-dominated at the early stage
3.0	0.404	0.230	0.545	0.719	0.110	0.736	Slow release under acidic conditions
1.2	0.792	0.185	0.861	0.861	0.101	0.854	Mainly diffusion-controlled release

The Korsmeyer–Peppas model was fitted using the initial release stage only.
An n value below 0.43 is generally considered consistent with Fickian diffusion for spherical systems.

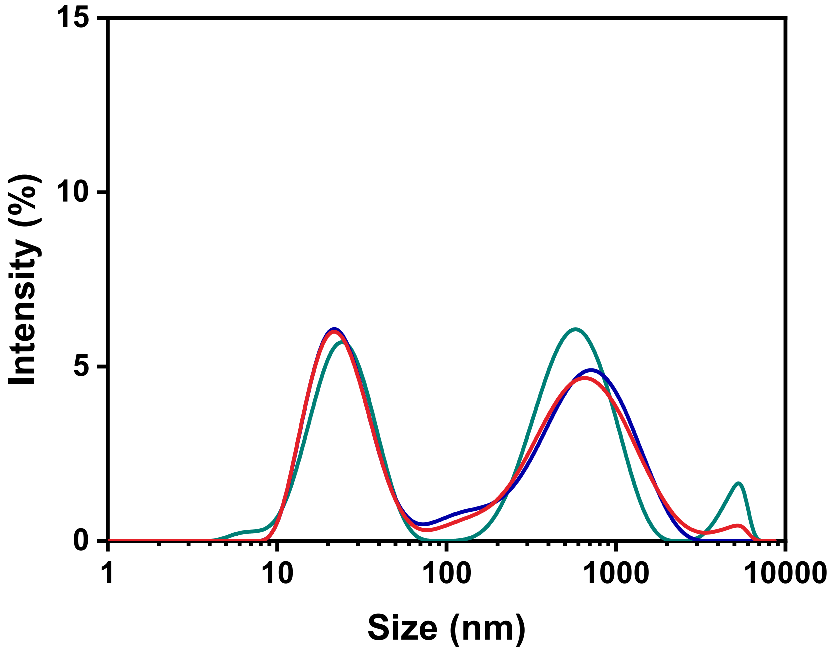

Figure S2. Particle size distribution of PLGA-NV-L (Parallel testing 3 times).

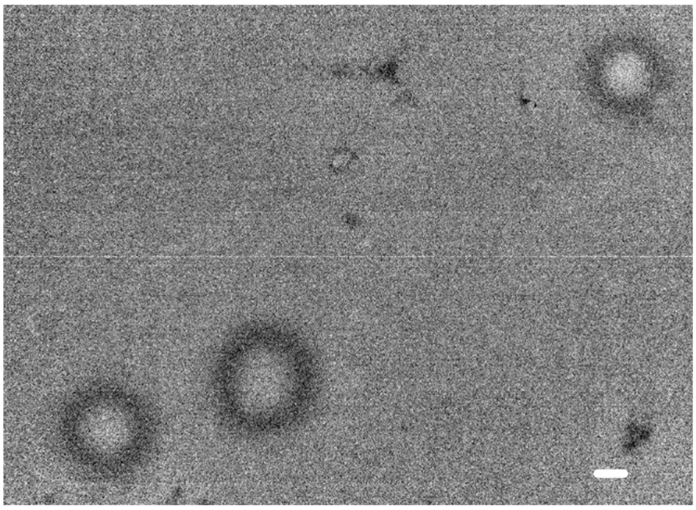

Figure S3.TEM image of PLGA-NV-L, the scale bar is 500 nm.
